# Association between ethylene oxide exposure and osteoarthritis risk among middle-aged and young adults: a cross-sectional study

**DOI:** 10.3389/fpubh.2025.1550456

**Published:** 2025-09-30

**Authors:** Xudong Wang, Meng Wang, Zijian Guo, Chuan Xiang

**Affiliations:** ^1^Department of Orthopedics, The Second Hospital of Shanxi Medical University, Taiyuan, Shanxi, China; ^2^Academy of Medical Sciences, Shanxi Medical University, Taiyuan, Shanxi, China

**Keywords:** osteoarthritis, ethylene oxide, middle-aged and young adults, cross-sectional study, national health and nutrition examination survey

## Abstract

**Background:**

The relationship between exposure to ethylene oxide (EO) and the risk of developing osteoarthritis (OA) remains unclear. We aimed to explore the association between EO exposure and OA risk among young and middle-aged adults.

**Methods:**

We utilized data from the 2013–2018 National Health and Nutrition Examination Survey, involving 2,380 individuals aged 20–60 years. Weighted multivariable regression models, smooth curve fitting (SCF), subgroup analysis and interaction tests were employed to examine the association between EO exposure and OA risk. Furthermore, we performed variable selection via least absolute shrinkage and selection operator regression and multivariable regression analyses to construct a prediction model.

**Results:**

Increased EO exposure was associated with increased OA risk. After full adjustment, individuals in the highest tertile of EO exposure had a significantly greater OA risk (224% increase) than did those in the lowest tertile of EO exposure (OR = 3.24; 95% CI: 1.61–6.52; *p* for trend = 0.002). SCF did not indicate any nonlinear associations. There was no statistically significant interaction observed in any of the subgroups (all *p* > 0.05). We built a prediction model visualized with a nomogram. This prediction model demonstrated good discriminatory power, excellent precision, and potential clinical benefits.

**Conclusion:**

The findings of our research demonstrated that among middle-aged and young adults, EO exposure was positively associated with OA risk. A prediction model was developed by integrating EO exposure with other factors readily acquired from users to assist in the evaluation and management of high-risk OA groups.

## Introduction

Osteoarthritis (OA) is the most common joint disease, and it is characterized by degenerative articular cartilage, altered synovium, and altered subchondral bone (1–3). OA is emerging as an increasing threat to public health. The number of Americans suffering from OA is expected to reach 67 million by 2030 (4). OA is one of the leading causes of chronic pain and prolonged disability among adults (5). Compared with the general population, OA patients have a greater propensity to experience psychological illnesses such as anxiety and depression (6–8). In addition to its impact on physical and mental health, OA also exerts a substantial economic influence on individuals and society (9–11). OA can affect individuals across all ages (6). Notably, young and middle-aged patients with OA tend to ignore their early symptoms and underestimate the serious consequences of the condition, leading to delays in diagnosis and treatment. Environmental toxins, which include heavy metals (like cadmium, lead) and air pollutants (like benzene), are likely to increase the risk of OA.

Ethylene oxide (EO), an industrial chemical, is widely employed as a sterilant for medical equipment and as an intermediate in the manufacture of other chemicals (12–14). EO exists in a gaseous state at room temperature, and inhalation is the principal route of exposure. Inhaled EO is readily absorbed into the bloodstream and can spread quickly throughout the body (15). The hemoglobin adduct of EO (HbEO) is produced by the binding of EO to Hb and has demonstrated excellent sensitivity and usefulness for evaluating EO exposure (16). People working in relevant fields are at high risk of EO exposure, while the general population may also inhale EO to a certain extent (17). Near sterilization facilities, the peak 24-h EO exposure of community inhabitants was far greater than that of individuals living in other areas (18). In addition, it is possible that EO exposure has increased with the occurrence of the COVID-19 pandemic and the increasing need for personal protective equipment (e.g., masks and gloves) sterilized with EO (19). Notably, EO is somewhat toxic, and overexposure to EO is extremely detrimental to human health. Nevertheless, the relationship between exposure to EO and OA risk remains unclear. There is increasing evidence that EO exposure is connected to inflammation and oxidative stress (20, 21). Since inflammation and oxidative stress play important roles in OA occurrence, we speculate that EO exposure may be closely correlated with OA risk.

In this study, we aimed to explore the association between EO exposure and OA risk among young and middle-aged adults.

## Methods

### Study population

**Figure 1:**
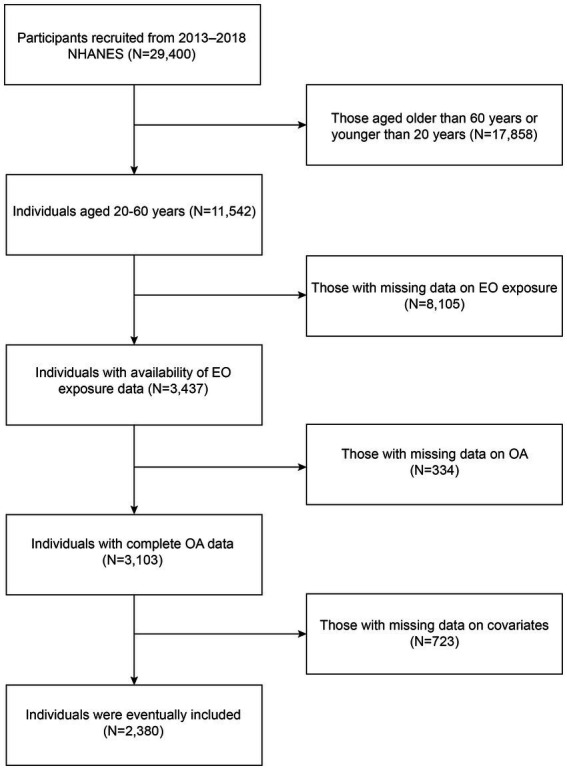
Flow chart of the study.

In the present study, we utilized cross-sectional data from the 2013–2018 National Health and Nutrition Examination Survey (NHANES). The NHANES is a nationwide program conducted by the National Center for Health Statistics (NCHS) to assess the nutritional and health conditions of Americans. Five types of data are included in the NHANES: demographic, examination, dietary, laboratory, and questionnaire data. The NCHS Research Ethics Review Board approved the entire program, and all participants provided written informed consent. The flowchart of the study is shown in Figure 1. Initially, a cohort of 29,400 participants from 2013 to 2018 NHANES were recruited. After excluding individuals older than 60 years or younger than 20 years (*N* = 17,858), those with missing data on EO exposure (*N* = 8,105), those with missing data on OA (*N* = 334), and those with missing data on covariates (*N* = 723), 2,380 individuals were eventually included.

### Assessment of OA status

The OA status of the participants was evaluated through a questionnaire. The participants were asked “Has a doctor or other health professional ever told you that you have arthritis?” Participants who answered “no” were considered not to have OA, and those who answered “yes” were then asked the following question: “What type of arthritis?” Participants who responded “osteoarthritis” were considered to have OA.

### Measurement of EO

HbEO has been established as a dependable biomarker for evaluating the level of EO exposure, since the half-life of HbEO *in vivo* is longer than that of EO. The participants’ morning blood samples were processed and preserved at −30 °C until they were sent to the National Center for Environmental Health for assessment. The quantity of HbEO was determined through a modified Edman reaction and high-performance liquid chromatography coupled with tandem mass spectrometry (HPLC-MS/MS). The outcomes of the measurements are presented as pmol/g Hb. Owing to the skewed distribution of HbEO levels, they were log2-transformed.

### Covariates

According to biological plausibility, prior related studies, and NHANES guidelines, the following covariate data were gathered: (1) demographic data: age (years), sex (male/female), race (Hispanic/non-Hispanic white/non-Hispanic black/other), education level (less than high school/high school/more than high school), and marital status (live with others/live alone); (2) examination data: body mass index (BMI, kg/m^2^); (3) questionnaire data: diabetes status (yes or borderline/no), hypertension status (yes/no), smoking status (smoked at least 100 cigarettes in life, yes/no), moderate activity status (at least 10 consecutive minutes of exercise or fitness or recreational activity during a typical week leading to a slight increase in respiration or heart rate, yes/no), and alcohol consumption status (drinking alcohol at least once per month, yes/no); and (4) laboratory data: albumin (g/dL), alanine transaminase (U/L), aspartate aminotransferase (U/L), blood urea nitrogen (mg/dL), total calcium (mg/dL), total protein (g/dL), high-density lipoprotein cholesterol (mg/dL), and total cholesterol (mg/dL).

### Statistical analysis

Statistical analysis was conducted utilizing appropriate NHANES sampling weights, accounting for the intricate multistage cluster survey. Continuous variables are represented as survey-weighted means [95% confidence intervals (CIs)], whereas categorical variables are displayed as survey-weighted percentages (95% CIs). We utilized weighted multivariable regression models to examine the association between EO exposure and OA risk. Model 1 remained unadjusted; Model 2 was adjusted for age, sex, and race; and Model 3 was adjusted for all covariates. The results are presented as odds ratios (ORs) and 95% CIs. We also employed smooth curve fitting (SCF) to explore the potential nonlinear associations between EO exposure and OA risk. Additionally, subgroup analysis and interaction tests were performed to assess the consistency of the associations across different subgroups. The stratification was based on age, sex, race, education level, marital status, diabetes status, hypertension status, moderate activity status, and alcohol consumption status.

We then selected the variables for constructing a prediction model via least absolute shrinkage and selection operator (LASSO) regression and multivariable regression analysis. The prediction model was visualized with a nomogram and evaluated via receiver operating characteristic (ROC) curves, calibration curves, and decision curve analysis (DCA). All analyses were conducted in R version 4.4.0^1^ and EmpowerStats.^2^ *p* < 0.05 was considered to indicate statistical significance.

## Results

### Baseline characteristics

The weighted demographic baseline characteristics of the study participants are depicted in Table 1. The participants were categorized into three groups according to the tertiles of EO exposure. Significant differences in race, education level, marital status, diabetes status, hypertension status, smoking status, moderate activity status, alcohol consumption status, OA status, BMI, blood urea nitrogen concentrations, total calcium concentrations, and high-density lipoprotein cholesterol concentrations (*p* < 0.05) were detected among the three groups.

**Table 1:** Baseline characteristics of participants, weighted.

Characteristics	EO exposure			*p*-value
	Tertile 1	Tertile 2	Tertile 3	
Age (years)	40.02 (39.17,40.87)	39.73 (38.39,41.06)	38.39 (37.24,39.55)	0.057
Sex (%)				0.051
Male	49.58 (45.03,54.14)	51.73 (47.59,55.84)	57.01 (52.72,61.20)	
Female	50.42 (45.86,54.97)	48.27 (44.16,52.41)	42.99 (38.80,47.28)	
Race (%)				<0.001
Hispanic	17.80 (13.79,22.67)	22.90 (18.09,28.55)	11.66 (8.94,15.08)	
Non-Hispanic white	68.34 (62.42,73.72)	55.33 (48.96,61.54)	61.20 (54.94,67.12)	
Non-Hispanic black	7.80 (5.63,10.70)	9.59 (7.15,12.74)	16.01 (12.67,20.04)	
Other	6.06 (4.42,8.26)	12.18 (9.60,15.33)	11.12 (7.77,15.69)	
Education level (%)				<0.001
Less than high school	5.79 (4.14,8.06)	10.57 (8.10,13.69)	19.28 (15.98,23.06)	
High school	18.15 (14.72,22.17)	19.58 (16.44,23.14)	31.67 (26.64,37.18)	
More than high school	76.06 (71.58,80.02)	69.85 (65.05,74.25)	49.05 (43.94,54.18)	
Marital status (%)				<0.001
Live with others	67.72 (63.27,71.87)	68.23 (63.34,72.75)	54.80 (49.82,59.68)	
Live alone	32.28 (28.13,36.73)	31.77 (27.25,36.66)	45.20 (40.32,50.18)	
Diabetes status (%)				0.038
Yes or borderline	9.32 (6.74,12.75)	10.60 (8.02,13.88)	6.07 (4.28,8.54)	
No	90.68 (87.25,93.26)	89.40 (86.12,91.98)	93.93 (91.46,95.72)	
Hypertension status (%)				0.007
Yes	21.20 (17.86,24.98)	20.00 (16.35,24.23)	27.84 (22.91,33.36)	
No	78.80 (75.02,82.14)	80.00 (75.77,83.65)	72.16 (66.64,77.09)	
Smoking status (%)				<0.001
Yes	28.92 (23.48,35.04)	30.20 (25.00,35.96)	82.63 (79.16,85.63)	
No	71.08 (64.96,76.52)	69.80 (64.04,75.00)	17.37 (14.37,20.84)	
Moderate activity status (%)				0.001
Yes	51.49 (45.78,57.15)	47.02 (42.27,51.82)	39.80 (35.28,44.50)	
No	48.51 (42.85,54.22)	52.98 (48.18,57.73)	60.20 (55.50,64.72)	
Alcohol consumption status (%)				0.019
Yes	70.17 (65.77,74.23)	63.89 (58.88,68.62)	61.75 (56.43,66.80)	
No	29.83 (25.77,34.23)	36.11 (31.38,41.12)	38.25 (33.20,43.57)	
OA status				0.007
Yes	6.45 (4.61,8.94)	11.77 (8.55,15.99)	12.81 (9.07,17.80)	
No	93.55 (91.06,95.39)	88.23 (84.01,91.45)	87.19 (82.20,90.93)	
BMI (kg/m^2^)	30.02 (29.30,30.75)	29.93 (28.98,30.89)	28.51 (27.89,29.13)	0.004
Albumin (g/dL)	4.27 (4.24,4.30)	4.29 (4.25,4.33)	4.28 (4.24,4.32)	0.331
Alanine transaminase (U/L)	26.38 (24.69,28.07)	26.52 (25.03,28.00)	28.09 (25.48,30.70)	0.598
Aspartate aminotransferase (U/L)	24.43 (23.41,25.44)	24.20 (23.30,25.10)	26.39 (24.36,28.43)	0.207
Blood urea nitrogen (mg/dL)	13.63 (13.25,14.00)	13.63 (13.09,14.17)	12.59 (12.16,13.01)	0.001
Total calcium (mg/dL)	9.32 (9.29,9.36)	9.36 (9.32,9.40)	9.39 (9.36,9.43)	0.001
Total protein (g/dL)	7.13 (7.09,7.16)	7.14 (7.10,7.18)	7.12 (7.07,7.17)	0.758
High-density lipoprotein cholesterol (mg/dL)	55.07 (53.56,56.58)	53.09 (51.27,54.91)	51.53 (50.22,52.84)	0.002
Total cholesterol (mg/dL)	187.50 (184.29,190.71)	189.93 (185.97,193.90)	187.87 (184.09,191.66)	0.566

*OA, osteoarthritis; BMI, body mass index*.

### Association between EO exposure and OA risk

**Figure 2:**
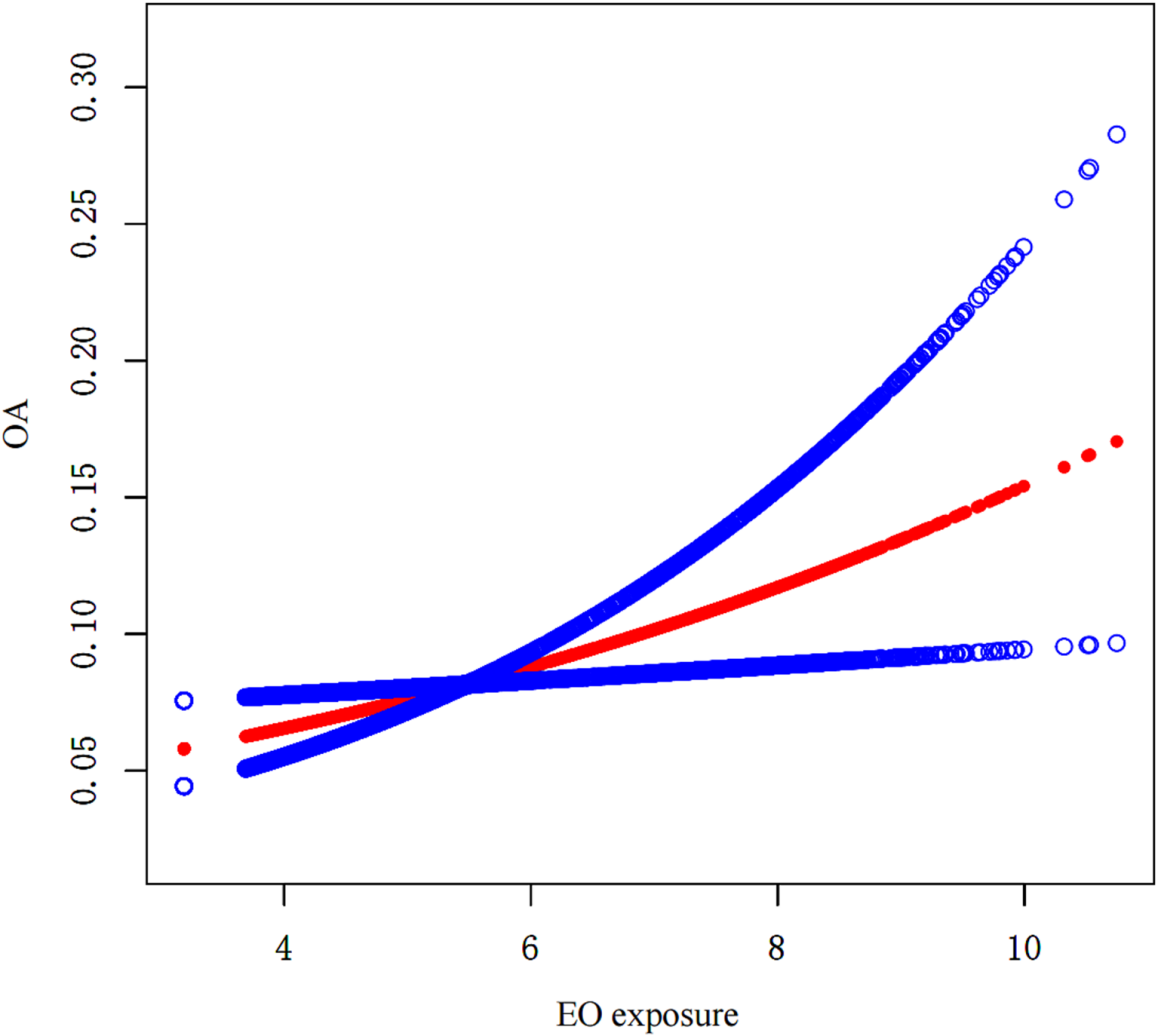
SCF analysis.

The association between EO exposure and OA risk is shown in Table 2. These findings indicated that increased EO exposure was related to increased OA risk. In both the crude and partially/fully adjusted models, a positive association was found between EO exposure and OA risk. After full adjustment, individuals with a one-unit rise in EO exposure had a 27% greater likelihood of developing OA (OR = 1.27; 95% CI: 1.09–1.47). Continuous EO exposure was then converted to a categorical variable on the basis of tertiles for sensitivity analysis. Compared with those in the lowest tertile of EO exposure, individuals in the highest tertile of EO exposure had a significantly greater OA risk, with a 224% increase (OR = 3.24; 95% CI: 1.61–6.52; *p* for trend = 0.002). Furthermore, the outcomes of SCF revealed that there was no nonlinear association between EO exposure and OA risk (Figure 2).

**Table 2:** Association between EO exposure and OA risk.

Characteristic	Odds ratio (95% CI)		
	Model 1	Model 2	Model 3
OA			
EO exposure (continuous)	1.16 (1.02, 1.31)	1.21 (1.07, 1.36)	1.27 (1.09, 1.47)
EO exposure (categories)			
Tertile 1 (cases/total = 50/790)	Reference	Reference	Reference
Tertile 2 (cases/total = 62/796)	1.94 (1.18, 3.19)	2.50 (1.44, 4.34)	2.51 (1.42, 4.45)
Tertile 3 (cases/total = 82/794)	2.13 (1.26, 3.60)	3.01 (1.68, 5.38)	3.24 (1.61, 6.52)
*P* for trend	0.005	<0.001	0.002

CI, confidence interval; OA, osteoarthritis; EO, ethylene oxide.

### Subgroup analysis

The results of the subgroup analysis and interaction tests are presented in Table 3. There was no statistically significant interaction observed in any of the subgroups (all *p* > 0.05), suggesting that the positive association between EO exposure and OA risk remained strong and consistent across different subpopulations.

**Table 3:** Subgroup analysis of the association between EO exposure and OA risk.

Characteristic	OA	OA
	OR (95% CI), *p*-value	*p* for interaction
Age		0.713
< 40	1.34 (0.94, 1.91) 0.124	
≥ 40	1.26 (1.08, 1.46) 0.006	
Sex		0.094
Male	1.40 (1.14, 1.72) 0.005	
Female	1.16 (0.98, 1.38) 0.099	
Race		0.192
Hispanic	0.98 (0.65, 1.48) 0.937	
Non-Hispanic white	1.34 (1.14, 1.58) 0.002	
Non-Hispanic black	1.07 (0.79, 1.45) 0.669	
Other	1.02 (0.71, 1.46) 0.925	
Education level		0.309
Less than high school	1.44 (1.11, 1.87) 0.013	
High school	1.07 (0.82, 1.39) 0.632	
More than high school	1.32 (1.08, 1.62) 0.013	
Marital status		0.639
Live with others	1.24 (1.05, 1.45) 0.017	
Live alone	1.31 (1.03, 1.68) 0.041	
Diabetes status		0.375
Yes/Borderline	1.45 (1.01, 2.08) 0.057	
No	1.24 (1.07, 1.44) 0.011	
Hypertension status		0.370
Yes	1.19 (0.98, 1.44) 0.088	
No	1.32 (1.10, 1.58) 0.007	
Smoking status		0.173
Yes	1.23 (1.06, 1.43) 0.013	
No	1.67 (1.06, 2.61) 0.037	
Moderate activity status		0.177
Yes	1.42 (1.10, 1.84) 0.015	
No	1.17 (1.00, 1.37) 0.069	
Alcohol consumption status		0.182
Yes	1.35 (1.11, 1.64) 0.007	
No	1.17 (0.99, 1.39) 0.077	

OA, osteoarthritis; OR, odd ratio; CI, confidence interval. All other covariates were adjusted, except the subgroup variable.

### Variable selection

**Figure 3:**
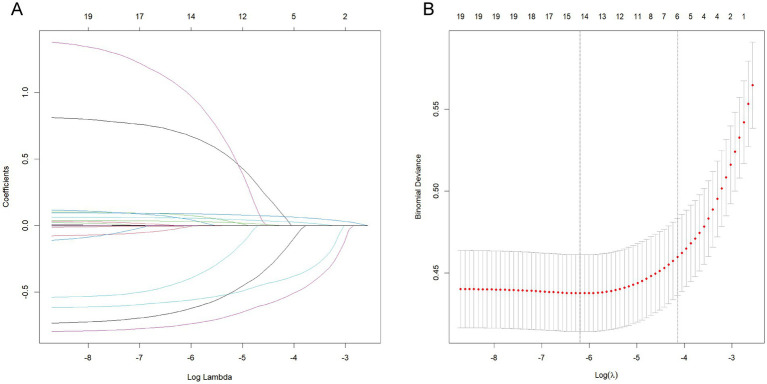
LASSO regression for related variables. **(A)** coefficient path plot. **(B)** cross-validation plot. The dashed lines on the left and right represent lambda.min and lambda.1se, respectively. We selected lambda.1se. The reason for choosing lambda.1se is that it leads to a more severe penalty and a lower number of variables than lambda.min does.

The prediction model initially considered candidate predictors that included indicators from the baseline characteristics in addition to EO exposure. Six key variables, including sex, diabetes status, hypertension status, smoking status, age, and BMI were selected through LASSO regression (Figure 3). The six selected variables were subsequently included in multivariable regression analysis to further screen the variables. The variables in that analysis with *p* values less than 0.05 were included in the prediction model (Table 4).

**Table 4:** Multivariable regression analysis for variable selection.

Variable	Odd ratio	95% CI-low	95% CI-upp	*p*-value
Intercept	0.0008	0.0002	0.0031	<0.0001
Sex (female)	1.7972	1.2902	2.5035	0.0005
Diabetes status (no)	0.5867	0.3923	0.8775	0.0094
Hypertension status (no)	0.4757	0.3387	0.6680	<0.0001
Smoking status (no)	0.5016	0.3606	0.6979	<0.0001
Age	1.0966	1.0766	1.1171	<0.0001
BMI	1.0488	1.0278	1.0703	<0.0001

CI, confidence interval; BMI, body mass index.

### Nomogram development for risk prediction

**Figure 4:**
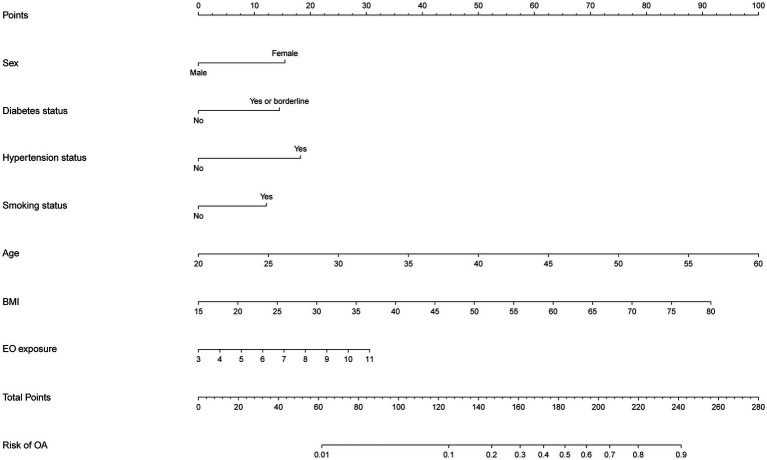
Nomogram for OA risk prediction.

After two stages of variable selection (LASSO regression and multivariable regression analysis), we built a prediction model that was visualized with a nomogram (Figure 4). The final prediction model incorporated seven predictors (EO exposure, sex, diabetes status, hypertension status, smoking status, age, and BMI). The nomogram consisted of 10 axes, where axes two through eight corresponded to every predictor included in the prediction model. Every predictor was given its own score in the nomogram. Axes nine and 10 showed that as the overall score increased, the OA risk increased accordingly.

### Assessment of the prediction model

**Figure 5:**
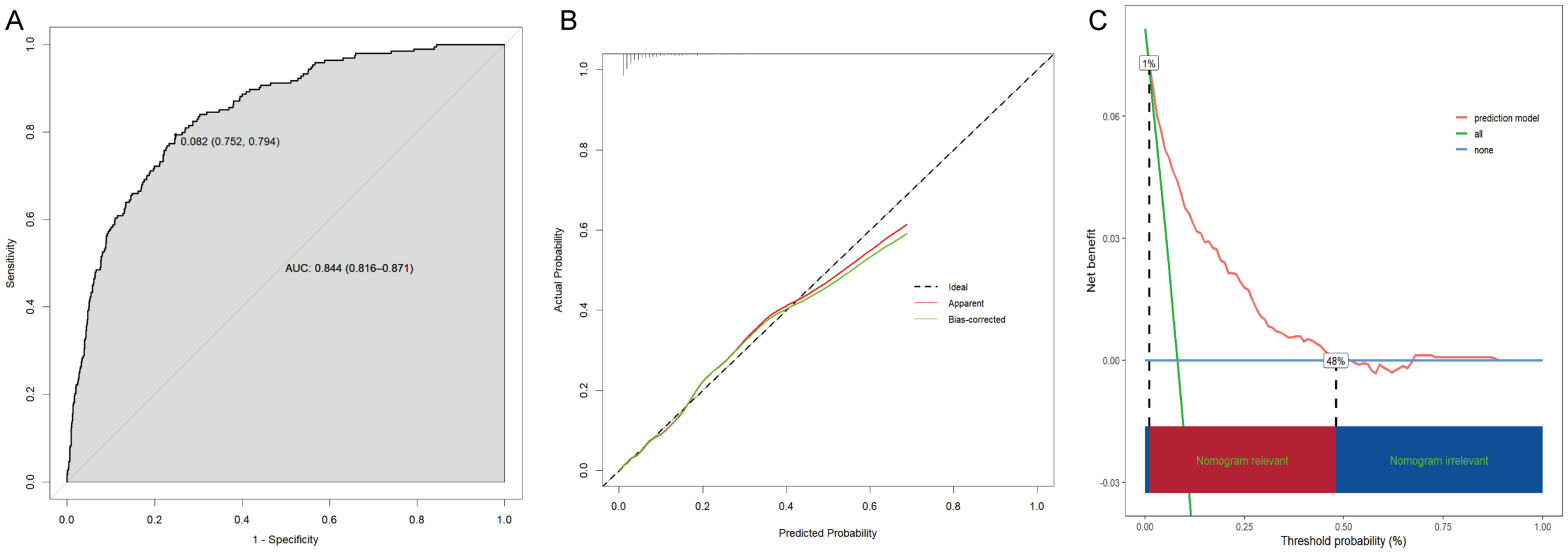
Assessment of the prediction model. **(A)** ROC curve. **(B)** Calibration curve. **(C)** DCA.

With an area under the ROC curve of 0.844 (95% CI: 0.816–0.871), the model clearly had good discriminatory power (Figure 5A). As illustrated in the calibration curve, the predicted probabilities were highly consistent with the actual probabilities, which indicates a high level of precision in the prediction model (Figure 5B). According to the DCA results, the prediction model was beneficial when the risk threshold was below 0.48 (Figure 5C).

## Discussion

According to the 2013–2018 NHANES, a total of 2,380 eligible young and middle-aged adults were recruited for our cross-sectional study. The main outcomes of this research were as follows. (1) EO exposure was shown to be positively correlated with OA risk. (2) The positive association between EO exposure and OA risk remained strong and consistent across different subpopulations. (3) We constructed a prediction model for OA risk by combining variables that could be readily acquired from users, including EO exposure, sex, diabetes status, hypertension status, smoking status, age, and BMI. (4) This prediction model demonstrated good discriminatory power, excellent precision, and potential clinical benefits. The above findings suggest that this model has the potential to be a straightforward and user-friendly instrument for assessing OA risk.

OA represents a slowly advancing condition impacting the human joint system (22). It can impact any synovial joint, with the hip, knee, hand, foot, and spine being the most often influenced locations (23). In addition to being painful, OA may also contribute to disability and reduce one’s life expectancy (24). Currently, the complicated etiology of OA is not completely known, but there is a growing consensus that nonignorable environmental factors may be responsible for this condition (25, 26). EO is widely present in the environment (13). It has a molecular weight of 44.05, is highly soluble in both water and organic solvents, and has the ability to react with a wide range of chemicals (27). The yearly output of EO is approximately 35 million metric tons, and the worldwide demand for EO is projected to increase the yearly output rate by a minimum of 2% by 2025 (28). EO can be emitted into the atmosphere in substantial amounts through industrial facilities, cigarette smoke, and automobile emissions, thereby contributing to environmental pollution (29–31). EO in the atmosphere can undergo degradation by reacting with photochemically generated hydroxyl radicals, but this process is slow (32). Industrial discharges of EO have been monitored by the U. S. Environmental Protection Agency since 1987 because of the potential adverse effects of EO exposure on human health (33).

The mutagenicity and carcinogenicity of EO have long been known. It has been proposed that EO induces genetic injury by the formation of DNA adducts, which leads to genetic mutations and chromosomal aberrations in both animals and humans (34). Multiple investigations have shown that prolonged EO exposure increases the likelihood of developing malignant neoplasms (such as leukemia, lymphoma, breast cancer, and gastric cancer) (15, 33, 35, 36). Prior studies have also examined the associations of EO with a variety of other illnesses or potentially unhealthy conditions (37–41). For example, Wu et al. reported that among nonsmokers, increased EO exposure was closely associated with elevated chronic kidney disease incidence and worse chronic kidney disease outcomes (37). Liu et al. reported that increased EO exposure was related to a decrease in cognitive function among older individuals (38). Additionally, a cross-sectional study involving 6,016 individuals revealed that elevated EO exposure was linked to increased susceptibility to depression, particularly in women, drinkers and smokers (39). In this study, EO exposure was positively related to OA risk. According to the sensitivity analysis, the OA risk was much greater for individuals in the highest tertile of EO exposure than for those in the lowest tertile. We observed a consistent trend across different subpopulations.

The pathogenesis of OA involves oxidative stress and inflammation (42, 43). Oxidative stress occurs when the formation of reactive oxygen species (ROS) exceeds the ability of the antioxidant defense system to remove them (44, 45). Oxidative stress is increased in OA chondrocytes and is a major cause of chronic inflammation (46–48). The levels of inflammatory mediators (such as IL-1β, TNF-*α*, and IL-6) are substantially elevated in OA joints, contributing to ROS generation (48). In other words, inflammation and oxidative stress are mutually dependent. They can activate signaling pathways in cartilage, generating phenotypic alterations marked by the failure of chondrocytes to maintain tissue homeostasis, thereby leading to OA (49–51). At present, the mechanism linking EO exposure to OA risk remains unknown. According to multiple studies, prolonged exposure to EO could result in diminished glutathione reductase activity and increased hepatic lipid peroxidation, both of which are linked to oxidative stress *in vivo* (52, 53). Chronic exposure to EO can also contribute to inflammatory lesions in rodent organs (54). EO exposure has been reported to be strongly linked to the onset of depressive symptoms, with inflammation serving as a crucial mediator (55). In addition, there is evidence that inflammation is involved in the occurrence of EO-associated periodontitis (56). Hence, it is plausible that increased EO exposure may increase OA risk via inflammation and oxidative stress.

Overall, our research revealed a positive association between EO exposure and OA risk among middle-aged and young adults. Inflammation and oxidative stress may be the mechanisms underlying this association, but additional verification is needed in subsequent investigations. Additionally, other factors, such as age, undoubtedly influence the occurrence of OA, as demonstrated by our multivariable regression analysis. Therefore, we included these readily obtainable factors related to the occurrence of OA in our prediction model to make it more effective and accurate, thereby contributing to the recognition and management of high-risk populations. Environmental factors are among the modifiable risk factors for OA. We advise that workers in related industries minimize EO exposure as much as possible or adopt appropriate safeguards to decrease professional risk. If an EO factory is surrounded by a heavily-populated community, it is advisable to relocate the factory to a less-populated area. Given the increased utilization of personal protective equipment in recent years, it is necessary to create a wholly innocuous sterilization substance to replace EO. For high-risk OA patients, especially those with high exposure to EO, anti-inflammatory therapies may be considered to prevent OA.

Notably, in the sex-stratified analysis, the association between EO exposure and OA risk was significant in males but not in females, although the *p* for interaction did not reach statistical significance. Regarding the potential mechanisms underlying subgroup differences, the researchers proposed the following explanations. Changes in enzyme activity because of sex differences may allow female systems to detoxify EO more rapidly (57). Furthermore, estrogen levels in females have a protective effect on cartilage, whereas males lack this effect (58, 59).

However, several limitations of this research must be acknowledged. Initially, although the concordance between self-reported and clinically diagnosed OA cases was high (81%) (60), determining whether a participant has OA based on the questionnaire may still lead to bias. Secondly, establishing a causal relationship between EO exposure and OA risk with a cross-sectional design was infeasible, necessitating further confirmation through a prospective cohort study. Similar to other epidemiological analyses, including each relevant covariate that may influence EO exposure or OA risk is difficult. Additional covariates, although absent from our current model, are potentially predictive and require further examination. Moreover, the prediction model requires validation in a larger external cohort. Finally, given that EO exposure can vary dynamically, a single measurement may fail to accurately represent the cumulative exposure and its effects on the risk of developing OA. Subsequent research should involve data from multiple measurements taken at different times to investigate the cumulative impacts of EO exposure on the risk of developing OA.

## Conclusion

The findings of our research demonstrated that among middle-aged and young adults, EO exposure was positively associated with OA risk. A prediction model was developed by integrating EO exposure with other factors that are readily acquired from users to assist in the evaluation and management of high-risk OA groups. There is no doubt that people should reduce their exposure to EO as much as possible. For high-risk OA patients, especially those with high exposure to EO, anti-inflammatory therapies may be considered to prevent the development of OA. Additional prospective research is needed to corroborate our findings.

## Data Availability

Publicly available datasets were analyzed in this study. This data can be found at: National Health and Nutrition Examination Surveys database (https://www.cdc.gov/nchs/nhanes/).

## Footnotes

^1^ http://www.R-project.org

^2^ www.empowerstats.com
